# Are teachers' and students' emotions reciprocally transmitted in the classroom?

**DOI:** 10.1111/bjep.70006

**Published:** 2025-06-27

**Authors:** Pei‐Hsin Li, Diane Mayer, Lars‐Erik Malmberg

**Affiliations:** ^1^ University of Oxford Oxford UK; ^2^ University of Helsinki Helsinki Finland

**Keywords:** emotion transmission, reciprocal relationship, student emotions, teacher emotions

## Abstract

**Background:**

Understanding teachers' and students' emotions in the classroom is acknowledged as beneficial for teaching and learning. While existing studies have emphasized the relationships between teachers and students, it remains unclear how teachers' and students' emotions are transmitted from one lesson to the next.

**Aims:**

We investigated the transmission of teachers' and students' emotions by exploring reciprocal relationships between their emotional experiences across lessons.

**Sample:**

Twenty homeroom teachers and their students in Grades 4–5 (*N* = 306) in Taiwan participated.

**Method:**

Using an intra‐individual design, we collected teachers' self‐reported lesson‐specific positive and negative emotions (4 items per composite; *n* = 249 lessons) and students' perceptions of teachers' emotions as well as their own emotions (*n* = 3884 lessons) at the end of each lesson over 5 days. We specified cross‐classified structural equation models (CCSEM).

**Results:**

Most variance in teachers' emotions was at the teacher level (ICC = .62), whereas students' negative emotions varied the most at the lesson level (ICC = .49–.52). Teachers' and students' prior emotions carried over to subsequent lessons (positive/negative: *β =* .14–.20/.17–.27). We found a reciprocal transmission of same‐valence emotions between students' perceptions of teachers' and their own emotions. Additionally, teachers' prior positive emotions were associated with students' positive emotions.

**Conclusions:**

The study supports a reciprocal relationship between students' perceptions of teachers' emotions and their own emotions, whereas teachers' emotions are linked primarily to their own previous emotions. These results suggest that enhancing emotional experiences in class requires consideration of both teachers' and students' emotions simultaneously.

## INTRODUCTION

In school settings, emotions, as psychological states, are essential outcomes and contributors to teaching and learning (Pekrun et al., 2017; Trampe et al., 2015). Consider the following fictitious scenarios. Ming, a primary school student, encounters a challenging math problem and feels upset. However, his teacher is busy teaching the materials and is expressing disappointment about the amount of time being spent on the topic. He appears tense and this causes Ming to feel upset at the end of the lesson. In the subsequent lesson with the same teacher, Ming is still feeling the frustration he experienced in the previous lesson, even though his teacher is now more relaxed.

Ya‐Ting is a homeroom teacher who teaches multiple subjects to a group of students and spends mornings, afternoons and lunchtimes with the group. She has read a funny joke, and she shares this with her students in one of the morning lessons. All the students giggle and laugh, and the lesson ends in a positive atmosphere. Some students discuss their happiness with this interaction with the teacher, whereas others share the joke with their friends in other classes during the break. This sets a light‐hearted tone for their next lesson, and Ya‐Ting feels enthusiastic.

These examples illustrate how teachers' and students' emotions can carry over from one lesson to the next, but also change during a school day. Teachers may perceive their students' emotions and adjust their teaching accordingly. Likewise, the class dynamics and teachers' emotions can trigger students' emotional responses. A teacher's positive emotions may enhance students' positive emotions or diminish their negative emotions. Conversely, a teacher's negative emotions, such as expressing disappointment and frustration, may decrease students' positive emotions and increase negative emotions.

Emotions usually refer to relatively brief feelings that arise in reaction to a specific situation or stimulus. It is believed that a person's positive and negative emotions can affect how they feel and may accumulate to shape behaviour over time (Fredrickson, 2001). Furthermore, emotions are not solely individual experiences; they unfold within a social context, potentially triggering the transmission of emotions between individuals (Herrando & Constantinides, 2021). Taken together, it seems that there is no consensus about the time span over which emotions are stable or fluctuate, nor about how certain emotions interact across time with other emotions. To investigate ‘carry‐over’ (the stability of one emotion over time) and ‘spill‐over’ (how one emotion can affect another emotion) within and between individuals over time, chronologically ordered data are necessary. Therefore, this study employs an intensive longitudinal design to investigate emotions in the naturally occurring pattern of school days, specifically the ‘carry‐over’ and ‘spill‐over’ from one lesson to the next.

To the best of our knowledge, this study goes beyond previous studies in four ways: First, we focus on state‐emotions (i.e., end‐of‐lesson reports of emotions), whereas most previous studies have focused on trait‐emotions (i.e., reports based on a single time‐point). Second, we collected both teacher and student‐reported state‐emotions, rather than relying on either students' or teacher's perspectives. Third, by specifying cross‐classified models, we were able to model multiple students' reports alongside one teacher report for each lesson. Fourth, while previous studies on teachers' and students' emotions have examined differences in emotions across various school subjects (Keller et al., 2025), we investigated how emotions ‘carried over’ and ‘spilled over’ from one lesson to another, and how emotions between teachers were transmitted and converged. In order to position our foci within the relevant literature, the following sections discuss emotions in academic settings, the phenomenon of emotion transmission and evidence of emotion transmission in classrooms.

### Emotions in academic settings

Emotions are seen as relatively brief states with multiple components related to a particular personally meaningful situation (Fredrickson, 2001; Pekrun, 2006; Schutz et al., 2006). Academic emotions refer to those experienced in academic settings, during learning and teaching activities (Pekrun et al., 2002). Emotions can be examined from either a discrete or a dimensional perspective. The discrete approach focuses on distinct emotions, such as enjoyment, anger and boredom (e.g., Frenzel et al., 2015, 2018), while the dimensional approach categorizes emotions by valence (i.e., positive and negative, Watson & Tellegen, 1985) or by both valence and activation, such as positive and negative deactivating emotions. According to Pekrun (2024), academic emotions can be categorized by valence (positive vs. negative), physiological arousal (activating vs. deactivating), object focus (activity vs. outcome), or across all three dimensions.

While research using discrete emotions in intraindividual studies employs single‐item indicators, valence‐based scales rely on multiple indicators supported by factor analyses (Crawford & Henry, 2004), and valence‐ and activation‐based scales are supported by circumplex models (Gurtman & Pincus, 2003). Among valence‐based measures, the distinction between positive and negative emotions is one of the most widely recognized ways for describing and communicating emotions (Watson & Tellegen, 1985). In line with the concept that positive and negative emotions may have different self‐perpetuating but interwoven systems in accumulation (Fredrickson, 2001; Harmon‐Jones et al., 2017), this study explored the interplay of positive and negative emotions between teachers and students across lessons.

Understanding students' emotions is central to understanding emotions in academic settings. Positive emotions, such as enjoyment, pride and relaxation, and negative emotions, such as anger, anxiety and boredom, are commonly observed in students (Pekrun et al., 2002). Previous studies indicate that students' emotions in academic settings (e.g., enjoyment, anger, boredom, frustration, anxiety) are associated with important educational outcomes, including self‐regulated learning and academic achievement (Camacho‐Morles et al., 2021; Pekrun et al., 2002, 2017).

It is also important to understand teachers' emotions. Research has shown that teachers experience a wide range of emotions in the classroom (Hargreaves, 2000; Prosen et al., 2011). These are often triggered by interaction with students and can impact both their instructional behaviour and students' emotions (Becker et al., 2014, 2015; Frenzel, 2014; Hagenauer et al., 2015). Notably, primary school homeroom teachers often experience more intense emotional involvement because they spend extended time with the same group of students (Hargreaves, 2000).

The optimal classroom climate would be one in which both teachers and students experience positive and activating emotions (Schutz, 2014). For this reason, it is important to investigate both teachers' and students' emotions in an academic context. Studies on student and teacher emotions can be grouped into single‐reporter studies and multiple‐reporter studies. Single‐reporter studies investigate either students' or teachers' reports of their own emotions as well as their perceptions of others' emotions. In multiple‐reporter studies, both students and teachers report on their own emotions as well as on perceptions of others' emotions. Although several studies have included both students' and teachers' emotions, most have focused on students as single reporters (e.g., Becker et al., 2014; Chang & Cherng, 2017; Keller & Becker, 2021). This study investigated emotions from multiple reporters (i.e., teachers and students).

### Emotion transmission in the classroom

The phenomenon of individuals transferring or ‘catching’ others' emotions in interactive contexts is ubiquitous. In the literature, various terms such as emotion transmission (Larson & Almeida, 1999), emotional contagion (Bolger et al., 1989) and emotional crossover (Westman et al., 2013) have been used to signify this phenomenon (Frenzel et al., 2009, 2018), encompassing both relatively unconscious and conscious processes. Consistent with Butler (2011), we adopt the term emotion transmission to describe the underlying process by which emotions transfer from one person to another, noting its time‐lagged nature. For example, if a teacher feels happy while teaching, students may catch this happiness in subsequent lessons. Although emotion transmission is typically viewed as valence‐specific (i.e., positive emotions lead to positive emotions but not negative emotions), previous research has indicated that cross‐valence transmission may occur and can be understood as a series of actions and reactions. For example, it may occur when a rise in one emotional experience leads to a decline in the opposite emotional state (e.g., positive emotions leading to a decrease in negative emotions) (Mancini et al., 2016).

The phenomenon of emotion transmission has been studied in various contexts, such as families (e.g., couples, parents and their children) and workplaces (e.g., psychotherapist‐client, leader‐staff), using approaches like observed facial mimicry and self‐reported emotions. With growing interest in emotions in the classroom, research on transmission phenomena has also included the micro‐level. For example, facial mimicry is considered an underlying mechanism in transmitting emotions, occurring within seconds that precede individuals' emotional states and perceptions. However, these micro‐interactions do not necessarily lead to students' subjective emotional experiences (Frenzel et al., 2024; Olszanowski et al., 2019). In contrast, self‐reported situational emotions focus on individuals' subjective experiences, perceptions and appraisals of a situation, and these may take time to develop and manifest in terms of transmission. These findings highlight the significant role of individual appraisal in the emotion transmission process and suggest that the timescale of this process can extend beyond immediate interactions.

Emotion transmission between teachers and students may be even more complex in the classroom setting. Teachers work with an entire group of students while simultaneously engaging both behaviourally and emotionally with individual students. For example, Li et al. (2024) showed that teachers who were more involved with individual students reported more positive emotions and fewer negative emotions when these particular students had higher engagement. To manage and regulate their emotions in line with school and societal expectations, teachers often use strategies such as surface acting (e.g., pretending to be happy or suppressing anger; de Ruiter et al., 2021; Glomb & Tews, 2004; Keller & Becker, 2021). Thus, although teachers may be the more influential party or senders of emotion transmission, it can be challenging for students to perceive their teachers' authentic emotions over time.

Previous findings on the relationships between teachers' and students' emotions provide insight into this area. For example, students' trait‐like self‐reported emotions may not be associated with teachers' self‐reported emotions (e.g., anxiety), or these effects may disappear when student‐perceived teacher emotions and teachers' emotional authenticity (e.g., enjoyment and anger) are taken into account (Keller & Becker, 2021). Similar findings were observed in concurrent in‐class boredom with teachers' self‐reported boredom not correlated with students' boredom (Tam et al., 2019). Instead, students' interpretations of their teachers' emotions appear to be strongly linked with their own emotions (Keller & Becker, 2021; Tam et al., 2019).

As research on emotion transmission in classrooms is still in its early stages, the mechanisms underlying teacher–student emotion transmission in primary schools require further investigation. Compared to secondary school settings, primary school teachers and students may experience greater emotional intensity and exchange due to their emotional and physical closeness and time spent together (Hargreaves, 2000; Prosen et al., 2011). The emotional bonds are likely stronger between homeroom teachers and their students due to the considerable time they spend together. Research suggests that emotion transmission may be selective, occurring mainly when the emotions are relevant to individuals (Delvaux et al., 2016). This indicates that discrepancies may exist between teacher‐reported and student‐reported emotional experiences in primary school due to factors such as the nature of emotion transmission and teachers' emotional self‐regulation. Given the limited research in primary schools, we investigated how positive and negative emotions are transmitted between primary school teachers and their students. Our study considered students' ability to understand these emotions by drawing on previous research (Pons et al., 2004) as well as observations of students' reactions in a pilot study of the present study.

### Reciprocal relationship between teachers' and students' emotions

Studies have shown mixed findings regarding how teachers' and students' emotions covary in school settings. For example, Chang and Cherng (2017) used a cross‐sectional survey and found that students' perceptions of teachers' trait‐like positive and negative emotions were associated with students' own emotions in secondary schools. Using the experience sampling method, Becker et al. (2014) reported that students' perceptions of their teachers' enjoyment, anger and anxiety during lessons were correlated with their own experiences of these emotions (*β* = .12–.27). In contrast, Tam et al. (2019) indicated that while student‐perceived teachers' boredom aligned with students' own boredom, teachers' trait‐like boredom during lessons was unrelated to students' ratings. These studies predominantly relied on concurrent emotion reports from a single informant (i.e., students), presenting a potential limitation in examining emotion transmission.

Although teacher–student emotion transmission is believed to be bi‐directional, empirical understanding of its mechanism remains limited, particularly in relation to time gaps and the inclusion of both teacher and student ratings. Two longitudinal studies shed light on this reciprocal process. Frenzel et al. (2009) found that students' trait‐like enjoyment in Grade 7 predicted teachers' enjoyment a year later, but students' enjoyment in Grade 8 was not correlated with teachers' concurrent enjoyment. Similarly, Frenzel et al. (2018) reported that students' trait‐like enjoyment at the start of the school year (Time1) predicted their own enjoyment at mid‐term (Time3). Furthermore, student enjoyment at Time 1 indirectly predicted teachers' enjoyment at mid‐term via teacher‐perceived student engagement (Time2, indirect effect: *β* = .21, *p* = .011). Student‐perceived teacher enthusiasm also partially mediated the relationship between teachers' enjoyment at Time1 and average student enjoyment at Time3. These findings suggest that emotions, particularly positive ones, can accumulate over time (Fredrickson, 2004), and that teacher–student emotion transmission operates on a larger timescale and in a mutual manner.

Reciprocal emotion transmission involves processes where a delayed effect occurs through either facial expression mimicry or individuals' evaluation of their emotions. However, little is known about teacher–student emotion transmission on a smaller timescale, particularly from the perspectives of both teachers and students. This study aimed to move beyond examining the concurrent association of teacher–student emotions during lessons to further validate existing assumptions and findings on reciprocal emotion transmission between teachers and students. To do this, we focused on investigating both teachers' and students' subjective emotional experiences throughout the lessons.

## THE PRESENT INTRA‐INDIVIDUAL APPROACH

This study examined the processes of teacher–student emotion transmission in primary school lessons, focusing on teachers' emotions, student‐perceived teachers' emotions and students' emotions across consecutive lessons. A unique design was employed to collect lesson‐specific emotional experiences from both teachers and students. This intra‐individual approach allows researchers to study the dynamics of variables across situations within individuals (Becker et al., 2014; Goetz et al., 2015; Tam et al., 2019). By enhancing contextual closeness, the method reduces disruptions to the teaching and learning process and minimizes retrospective biases (Bolger & Laurenceau, 2013).

**FIGURE 1:**
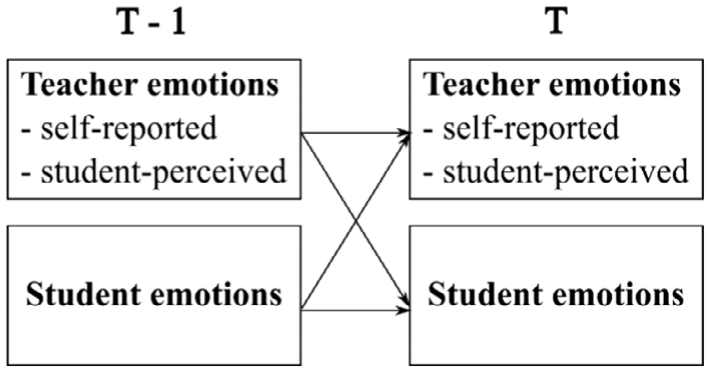
A framework for the reciprocal teacher–student emotion transmission.

In order to address gaps in the field of teacher–student emotion transmission, we proposed a conceptual framework (see Figure 1) to examine three aspects: the variance of positive and negative emotions in a specific cultural context; the spill‐over of emotions from one lesson to another; and the reciprocal emotion transmission between teachers and students across lessons. The following research questions were formulated along with corresponding hypotheses.

### RQ1: What is the proportion of variance at the within‐level (lessons) and between‐levels (students and teachers) of positive and negative emotions?

Our first objective was to explore the variance in emotional reports of teachers and students. Building on findings from Li et al. (2022, 2024), we hypothesized a larger proportion of variance in teacher emotions at the between‐teacher level in Taiwanese classrooms (Hypothesis 1.1). For students, previous research indicates that student‐reported emotional variability is largely attributable to between‐lesson variations (Becker et al., 2014; Goetz et al., 2020; Mainhard et al., 2018; Tam et al., 2019). Accordingly, we hypothesized that between‐lesson differences will primarily explain student emotions (Hypothesis 1.2).

### RQ2: What is the stability of students' and teachers' emotions over time?

Our second objective was to investigate the stability of teachers' self‐reported emotions, student‐perceived teachers' emotions and students' emotions over subsequent lessons. Drawing from previous research suggesting that trait‐like emotions tend to exhibit a stable trend over time with medium effect sizes (e.g., Frenzel et al., 2009, 2018), we hypothesized that the emotions of teachers and students would demonstrate weak to medium stability across lessons (Hypothesis 2).

### RQ3: Do same‐valence emotions transmit between teachers and students?

Our third objective was to examine the reciprocal relationships between (1) student‐perceived teachers' emotions and students' emotions and (2) teachers' self‐reported emotions and students' emotions. We examine the reciprocal relationships by analysing cross‐lagged effects. We were interested in relationships where teachers' emotions at a timepoint predict students' emotions at a later timepoint or vice versa. Previous research has consistently shown a concurrent association between student‐perceived teachers' emotions and students' self‐reported emotions (Becker et al., 2014; Chang & Cherng, 2017; Mottet & Beebe, 2000). However, findings are mixed related to the associations between teacher self‐reported emotions and students' self‐reported emotions, with some studies reporting significant concurrent relationships and others finding negligible associations (Keller & Becker, 2021; Tam et al., 2019). Expanding previous research on concurrent relationships, we hypothesized that student‐perceived teachers' emotions would be positively associated with same‐valence student emotions across lessons (Hypothesis 3.1). Conversely, we anticipated that the same‐valence cross‐lagged effects between teachers' self‐reported and students' emotions may not be uniformly present, with positive emotions likely to be more pronounced (e.g., Hubbard et al., 2023; Lin et al., 2024) (Hypothesis 3.2).

### RQ4: What is the relationship of cross‐valence emotions between teachers and students across lessons?

Our fourth objective was to explore whether cross‐valence emotions between teachers and students (e.g., teachers' previous positive emotions predict students' current negative emotions) are linked across lessons. Cross‐valence emotion transmission refers to the effect of one actor's previous positive emotions on another's negative emotions, or the effect of one actor's previous negative emotions on another's positive emotions. Due to the exploratory nature of this question and the limited prior evidence, we did not propose explicit hypotheses.

## METHOD

### Participants

A sample of 20 homeroom teachers in Taiwan (18 females; age range, 28–55) and their 306 students (144 girls) from grades 4 and 5 were included in the current study. Two students were excluded from the analysis due to lack of complete responses. Students' average age was 10.4 (SD = .67) and their average participation rate across classrooms was .64 (The participation rate in each class is provided in the Appendix A). The data were collected from eight public primary schools during an academic year in Taiwan and targeted lessons taught by homeroom teachers, who teach multiple subjects to the same group of students, such as Mandarin, Mathematics and Integrative Activities (see Table 1 for details).

**TABLE 1:** Characteristics of participants.

	*N*	%	Mean (SD)	Range
**Teachers (*N* = 20)**				
Gender				
Male	2	10.0		
Female	18	90.0		
Age	20	100.0	43.05 (9.01)	28–55
Teaching experience	20	100.0	16.13 (8.56)	2–30
**Students (*N* = 306)**				
Gender				
Boy	162	52.9		
Girl	144	47.1		
Age	304	99.3	10.40 (.67)	9.08–12.25
Family SES				
High	106	34.6		
Middle‐high	80	26.1		
Middle	103	33.7		
Middle‐low	12	3.9		
Low	0	0		
Mathematics	305	99.7	81.50 (14.84)	17–100
Mandarin	305	99.7	86.75 (10.38)	43–100

### Procedure

Teachers and students participated in the research over a 5‐day period. Informed consent was obtained from the teachers and from the parents and guardians of the students. Participants provided demographic information (e.g., gender, age) and completed paper‐and‐pencil questionnaires about their general well‐being at both the beginning and the end of the study (see also Li et al., 2022). At the end of each lesson, teachers and students completed a short questionnaire using tablets. Teachers self‐reported their emotions, and students reported their emotions as well as their perceptions of teachers' lesson‐specific emotions.

A total of 249 lesson reports from 20 teachers (M_responses_ = 12.45, SD = 2.09, Range = 8–16) and 3884 lesson reports from 306 students (M_responses_ = 12.69, SD = 2.27, Range = 5–16) were included to generate the merged data set and lagged variables.^1^ In general, teachers' diary entries per day ranged from 1.6 to 3.2 (M = 2.49, SD = .42), and students' diary entries per day ranged from 1.2 to 4.0 (M = 2.63, SD = .40), with the time span ranging from .37 to 6.18 h within days (M = 1.87, SD = 1.45). The median response time for teachers was 147 s (2 min 27 sec) and 83 s (1 min 23 s) for students.

### Measures

We included four positive (i.e., enjoyment, pride, relaxation, calmness) and four negative (i.e., anger, anxiety, disappointment, boredom) lesson‐specific emotions for both teachers and students (see Li et al., 2022). We selected these emotions based on previous research in classroom settings (e.g., Goetz et al., 2013; Pekrun et al., 2002, 2017) and on the findings of our pilot study on culture‐specific emotions among students and teachers in Taiwan.

**FIGURE 2:**
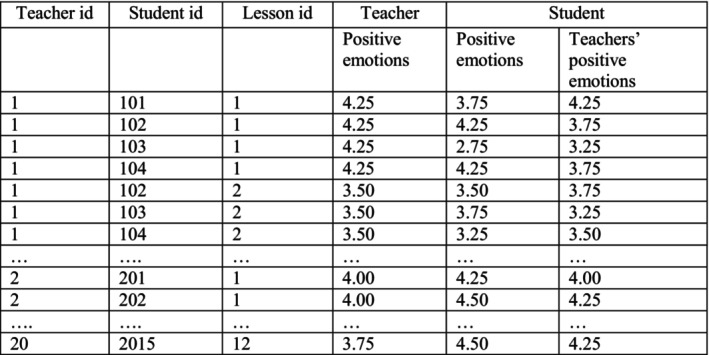
Schematic data set structure.

Teachers and students responded to items with wording such as, ‘In this lesson, I… felt enjoyment/was proud of myself/felt relaxed/was calm/felt angry/felt nervous/was bored/was disappointed’ on a 5‐point Likert scale (1 = not at all, 5 = very much). Student‐perceived teacher emotions were measured by a parallel item wording ‘In this lesson, my teacher… (e.g., was relaxed)’ with the same four positive emotions (enjoyment, pride, relaxation, calmness) and negative emotions (anger, anxiety, disappointment, boredom; 1 = not at all, 5 = very much). Reliabilities [Spearman‐Brown level‐2 reliability, average internal consistencies (Cronbach's *α*) across time‐segments and McDonald's] were all appropriate as indicated in Table 2. Furthermore, the cross‐classified confirmatory factor analysis (CCCFA) supported the appropriateness of the two‐factor structure (i.e., positive and negative emotions) in this study (for analysis details of CCCFA models for two‐factor and discrete emotions, please see Figures 1 and 2 in Appendix S1).

**TABLE 2:** Intercorrelations and descriptive statistics of research variables.

	(1)	(2)	(3)	(4)	(5)	(6)	(7)	(8)	(9)	(10)	(11)	(12)
Situation												
(1) SPE												
(2) SNE	**−.18**											
(3) STPE	.**60**	−.02										
(4) STNE	−.02	.**47**	.02									
(5) TPE	.03	**−.04**	.**07**	**−.07**								
(6) TNE	−.02	.**04**	**−.06**	.**09**	**−.37**							
(7) SPE(T−1)	.**23**	.**05**	.**19**	.**06**	**−.07**	.01						
(8) SNE(T−1)	.00	.**24**	.04	.**21**	.04	−.02	**−.24**					
(9) STPE(T−1)	.**20**	.**07**	.**26**	.**08**	**−.07**	.00	.**80**	**−.15**				
(10) STNE(T−1)	−.03	.**24**	.00	.**23**	.04	.01	**−.14**	.**65**	**−.16**			
(11) TPE(T−1)	.**06**	−.02	.**08**	**−.07**	.**15**	**−.13**	.**14**	**−.08**	.**19**	**−.10**		
(12) TNE(T−1)	.01	.01	−.03	.**05**	**−.14**	.**33**	**−.06**	.04	**−.12**	.**06**	**−.66**	
Student												
(13) SPE(S)												
(14) SNE(S)	**−.28**											
(15) STPE(S)	.**94**	**−.26**										
(16) STNE(S)	**−.24**	.**85**	**−.27**									
(17) TPE(S)	.**57**	−.37	.**58**	−.39								
(18) TNE(S)	−.15	.15	−.17	.11	−.33							
Teacher												
(19) SPE(S)												
(20) SNE(S)	**−.78**											
(21) STPE(S)	.**98**	−.71										
(22) STNE(S)	**−.87**	.**89**	**−.87**									
(23) TPE(S)	.55	−.37	.52	−.35								
(24) TNE(S)	−.39	.26	−.41	.26	−.81							
M	3.40	1.73	3.33	1.73	3.63	1.79	3.38	1.74	3.31	1.75	3.65	1.81
SD	.96	.83	.94	.84	.62	.69	.95	.82	.94	.84	.62	.70
Skewness	−.25	1.28	−.11	1.20	−.16	.45	−.24	1.28	−.11	1.18	−.11	.42
Kurtosis	−.34	1.77	−.30	1.35	.23	−.79	−.29	1.88	−.28	1.30	.15	−.85
ICC(W)	.42	.52	.36	.49	.38	.37						
ICC(S)	.47	.41	.49	.44	.002	.002						
ICC(T)	.10	.07	.15	.07	.62	.62						
ICC2(S)	.94	.91	.95	.92								
ICC2(T)	.71	.66	.78	.64	.94	.93						
Cronbach's *α*	.66	.74	.68	.81	.75	.81						
ω (W)	.61	.63	.56	.72	.59	.67						
ω (S)	.78	.91	.80	.93								
ω (T)					.88	.92						

*Note*: Bold values represent significant results. *N*_situation_ = 4073, *N*_student_ = 306, *N*_teacher_ = 20. ω (W)/ω (S)/ω (T) = McDonald's omega at different levels.

Abbreviations: Cronbach's *α*, average internal consistencies across time‐segments; ICC(S), student‐level between‐level intraclass correlation (from three‐level model); ICC(T), student‐level between‐level intraclass correlation (from three‐level model); ICC(W), within‐level intraclass correlation; ICC2(S), Spearman‐Brown level‐2 reliability at the student level; ICC2(T), Spearman‐Brown level‐2 reliability at the teacher level; SPE(T−1)/SNE(T−1), lagged student positive/negative emotions based on raw data; SPE/SNE, student positive/negative emotions; STPE/STNE, student‐perceived teacher positive/negative emotions; TPE/TNE, teacher positive/negative emotions.

### Analytical procedures

Given that our data were structured as lesson reports (Level 1: *N* = 3884) nested both within students (Level 2a: *N* = 306) and teachers (Level 2b: *N* = 20), cross‐classified models were specified and analysed using Mplus 8.4 (Muthén & Muthén, 1998–2017). Notably, the teacher reports were the same for each lesson for all the students, whereas student reports of the same lesson differed as a teacher shared the same lesson with multiple students (see Figure 2 for an example data set).

All models were estimated using a multilevel structural equation modelling (MSEM) framework with Bayesian estimation, applying admissible‐range‐restricted priors for variances (McNeish, 2019). Predictors at the lesson level were analysed using group mean centring to address the potential cluster dependency (Sanders & Konold, 2023). We first examined cross‐classified variance component models to estimate the variance in students' and teachers' positive and negative emotions.

To answer our first research question on variance proportions, we investigated the ICCs at the different levels. For our second research question on the stability of emotions over time, we regressed students' and teachers' emotions (at Time T) on their previous emotions (at Time T−1) at the lesson level, for positive and negative emotions separately. We interpreted the strength of the auto‐regressive coefficients as follows: a strong positive effect indicates inertia, suggesting that emotions would remain stable over time and return slowly to the average level; a weak effect suggests a quick return to the average emotion, meaning changes are relatively short‐lived; and, a negative effect indicates a ‘saw‐toothed’ patterns over time, where a high level of an emotion predicts a low level in the subsequent lesson, and vice versa.

To investigate Research Questions 3 and 4 on reciprocal effects between (1) teacher and student‐perceived teachers' emotions and (2) teacher emotions and student emotions, we inspected cross‐lagged effects at the lesson level (Beretvas, 2011; Fielding & Goldstein, 2006). We included students' performances (average of Mandarin and Math) as covariates in our models, as no relationships were found with the other covariate variables (i.e., students' gender, age, performance) in the initial analysis of stability models. Model outputs from Mplus are available at [https://osf.io/r6xzp/?view_only=b19f48c6d7f34082b1075246614dcf13].

Our cross‐classified MSEM with auto‐regressive and cross‐lagged paths at the within‐level is consistent with elements of the Vector Auto Regressive (VAR) model (Ariens et al., 2020), incorporated in the dynamic SEM module in Mplus (Hamaker et al., 2018), enabling latent lagged variable modelling, adjustment of non‐equidistant time‐lags and implementation of the Bayesian estimator. The Bayesian estimator enables estimations of complex models, also in multilevel data with sparse data at higher levels (Hox et al., 2012; McNeish, 2019). However, after exploring random slope models, we found limited individual differences in the auto‐regressive and cross‐lagged paths. We did not implement the latent lagged variables together with the adjusted non‐equidistant time‐lags, as this procedure is not (yet) possible in the cross‐classified model (In the Appendix S1, we present an example of a two‐level model with latent lags and adjusted time‐lags, which replicated the findings of the cross‐classified model). Hence, we adopted a modelling strategy in which the first time point of the lagged variable each day (i.e., the value of the last lesson the previous day is carried forward) was coded missing. This would account for the downtime between the last lesson on day D‐1 and day D (see e.g., Malmberg & Martin, 2019).

We specified semi‐informative priors and admissible‐range restricted priors for variance components following McNeish (2019). We set the Bayesian estimator at 50,000 iterations, using two chains and a thinning factor of 10. The quality of convergence and model fit was checked through posterior trace, autocorrelation, distribution plots and the maximum Potential Scale Reduction <1.05 (Asparouhov & Muthén, 2020).

## RESULTS

### Descriptive statistics

Teachers' emotions, student‐perceived teachers' emotions and students' self‐reported emotions showed a similar pattern in mean levels (see Table 2). Students' positive and negative emotions were significantly related to student‐perceived teachers' emotions. Specifically, positive emotions among students were higher when they perceived their teachers had higher positive emotions (*r* = .60) and when they experienced more positive emotions (*r* = .23) in the previous lesson. Students experienced negative emotions when they perceived teachers had stronger negative emotions (*r* = .47) and themselves had stronger negative emotions (*r* = .24) in the previous lesson. The teachers' positive and negative emotions were carried over from their own previous emotions of the same valence to the subsequent emotions (positive emotions at T and T−1: *r* = .15, negative emotions at T and T−1: *r* = .33 at the situation level).

### What is the variance in teachers' and students' emotions at the lesson level (RQ1)?

The intraclass correlation coefficients (ICCs) at different levels were calculated based on cross‐classified (timepoints nested in both students and teachers) models (see Table 2). The ICCs of student emotions indicated that most of the variance in students' emotions was attributed to situational variation for negative emotions (ICC_lesson_ = .52) and attributed to the student level for positive emotions (ICC_student_ = .47), partly supporting our first hypothesis.

A similar pattern was found for student‐perceived teachers' emotions. The ICCs showed that most of the variance in student‐perceived teachers' positive emotions originated at the student level (ICC_student_ = .49), while most of the variance in student‐perceived teachers' negative emotions originated at the lesson level (ICC_lesson_ = .49, ICC_student_ = .44). Interestingly, despite the substantive variance in teachers' self‐reported emotions across lessons, most of the variance originated from individual variation between teachers (ICC_teacher_ = .62 for both positive and negative emotions).

### What is the stability of students' and teachers' emotions over time (RQ2)?

**FIGURE 3:**
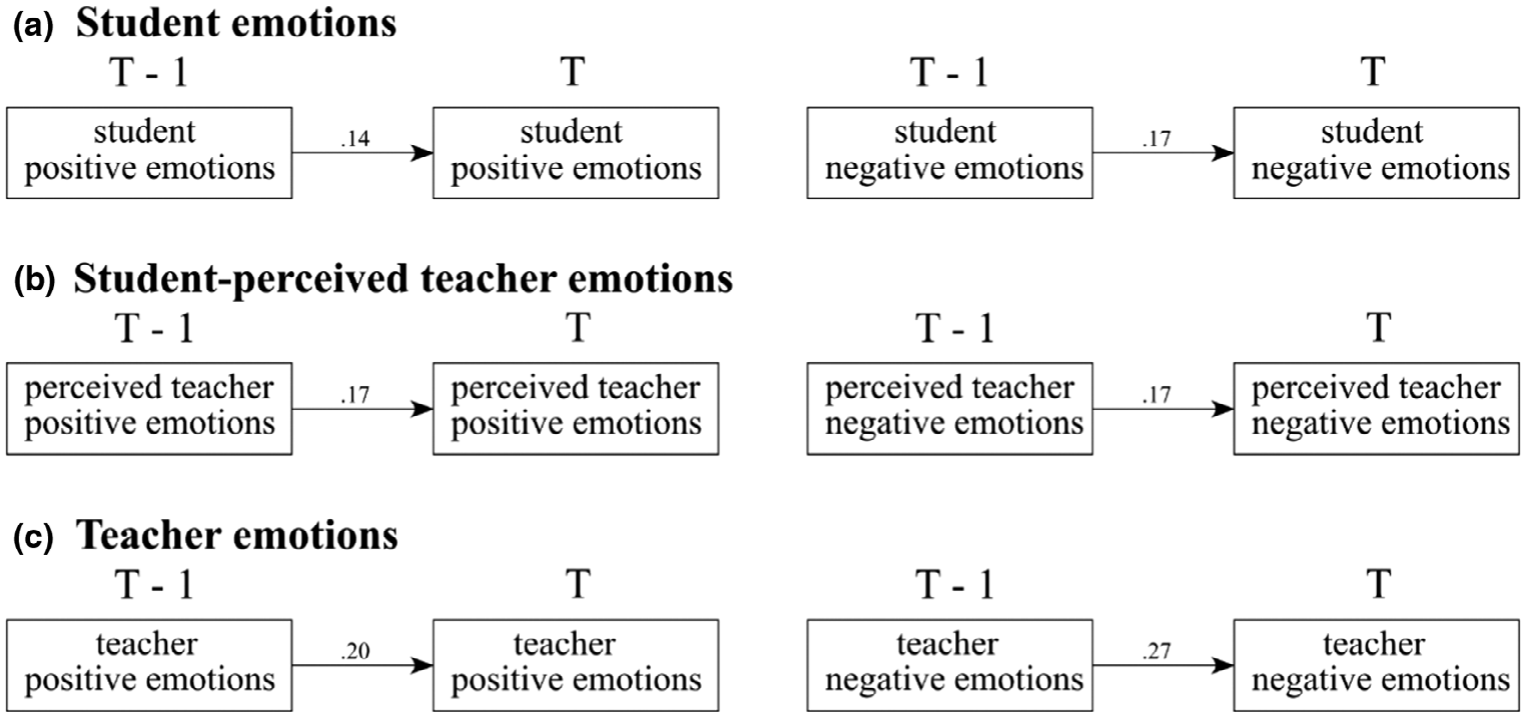
Teacher and student emotion stability. Standardized estimates were presented in the figure. The solid lines indicated credible intervals (CI) that do not include zero.

We first investigated the lagged effects of students' emotions, student‐perceived teachers' emotions and teachers' emotions, respectively. In accordance with Hypothesis 2, we examined whether teachers' and students' emotions demonstrated a weak to medium stability across lessons (see Figure 3).

Figure 3a illustrates the stability of students' emotional states across lessons. The autoregressive paths showed that students' previous positive emotions (T−1) predicted their current (T) positive emotions (*β* = .14, CI [.10, .18]). Similarly, students' previous negative emotions (T−1) predicted their subsequent negative emotions (*β* = .17, CI [.13, .20]). The autoregressive parameters were relatively weak, indicating that emotions returned to baseline (i.e., individuals' own average emotion) quite quickly, suggesting that the effects were short‐lived.

Figure 3b presents the stability of the student‐perceived teachers' emotions. The autoregressive paths showed that student‐perceived teachers' positive emotions in previous lessons predicted teachers' current positive emotions (*β* = .17, CI [.13, .21]). Student‐perceived teachers' negative emotions in previous lessons predicted teachers' current negative emotions (*β* = .17, CI [.13, .20]).

Figure 3c shows the autoregression of teacher emotions. Teachers' previous positive emotions (T−1) predicted their current positive emotions (*β* = .20, CI [.15, .24]). Similarly, teachers' previous negative emotions (T−1) predicted subsequent negative emotions (T) (*β* = .27, CI [.22, .31]).

### Do same‐valence emotions transmit between teachers and students (RQ3)?

#### Perceived teacher emotions and student emotions

**FIGURE 4:**
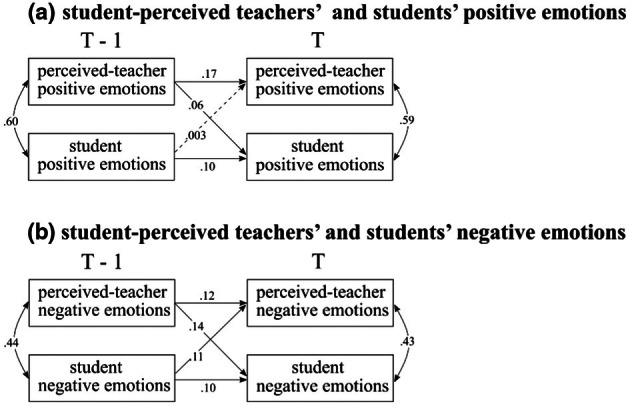
Transmission of same‐valence student‐perceived teachers' emotions and students' emotions. Standardized estimates were presented in the figure. Dotted lines represent zero is included in the credible interval (CI).

In order to investigate possible reciprocal transmission between student‐perceived teachers' emotions and students' emotions (Hypothesis 3.1), we examined the effects of student‐perceived teachers' positive emotions on students' positive emotions and vice versa (see Figure 4a). The effect of previous student‐perceived teachers' positive emotions on subsequent students' positive emotions was found (*β* = .06, CI [.02, .11]), whereas the effect of previous student positive emotions on current student‐perceived teachers' emotions was not found (*β* = .003, CI [−.05, .05]). At each timepoint, students' perceived teachers' positive emotions were correlated with their positive emotions (at T−1: *ρ* = .60, CI [.57, .62]; at T: *ρ* = .59, CI [.57, .61]).^2^

Following the same steps, we investigated the relationships between student‐perceived teachers' negative emotions and students' negative emotions across lessons (Figure 4b). The model showed that higher levels of previous student‐perceived teachers' negative emotions predicted higher current students' negative emotions (*β =* .14, CI [.10, .19]). Similarly, higher previous students' negative emotions predicted higher current student‐perceived teachers' negative emotions (*β =* .11, CI [.06, .15]). Additionally, concurrent relationships were observed between student‐perceived teachers' negative emotions and their negative emotions at both previous (T−1: *ρ* = .44, CI [.41, .47]) and current (T: *ρ* = .43, CI [.40, .46]) situations.

#### Teachers' and students' self‐reported emotions

**FIGURE 5:**
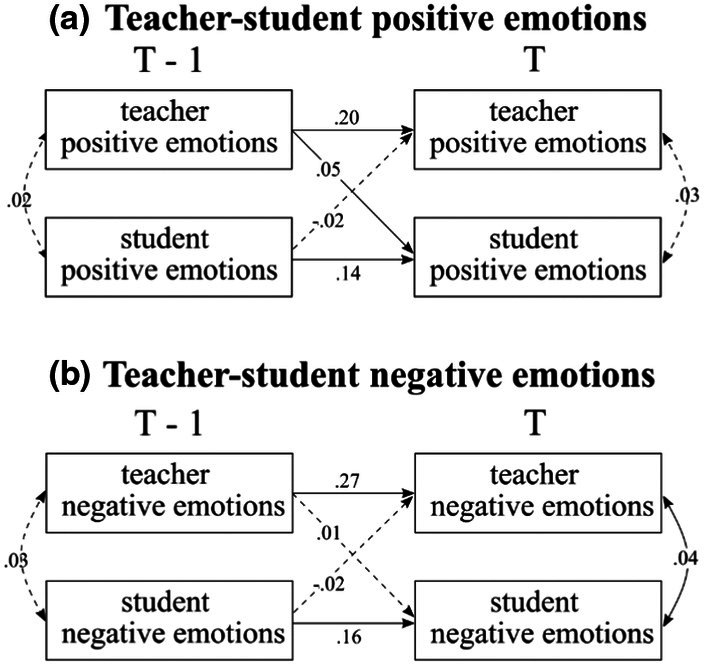
Transmission of same‐valence teacher‐reported emotions and students' emotions. Standardized estimates were presented in the figure. Dotted lines represent zero is included in the credible interval (CI).

Next, we examined the transmission between teachers' self‐reported and students' emotions across lessons (Hypothesis 3.2), following the same analytical steps as in the previous models. As Figure 5a shows, previous teachers' positive emotions predicted students' current positive emotions (*β* = .05, CI [.01, .09]). In contrast, students' positive emotions in the previous lessons showed no credible effects on teachers' current positive emotions (*β* = −.02, CI [−.06, .02]). Furthermore, no concurrent relationships were observed between teachers' and students' positive emotions (previous: *ρ* = .02, CI [−.02, .06]; current: *ρ* = .03, CI [−.004, .06]).

In terms of negative emotions (Figure 5b), no effect was found of previous teachers' negative emotions on current students' negative emotions (*β* = .01, CI [−.03, .05]) or vice versa (*β* = −.02, CI [−.06, .02]). Additionally, while no concurrent relationships were observed between previous (T−1) teachers' and students' negative emotions (*ρ* = .03, CI [−.01, .07]), a small but credible effect was found between current (T) teachers' and students' negative emotions (*ρ* = .04, CI [.01, .08]).

### What is the relationship of cross‐valence emotions between teachers and students across lessons (RQ4)?

We conducted two extended models to address our fourth research question regarding cross‐valence emotion transmission across lessons. First, we present the results concerning the relationships between student‐perceived teachers' emotions and students' emotions. Subsequently, we present the results concerning the relationship between teachers' emotions and students' emotions.

#### Perceived teacher emotions and student emotions

**FIGURE 6:**
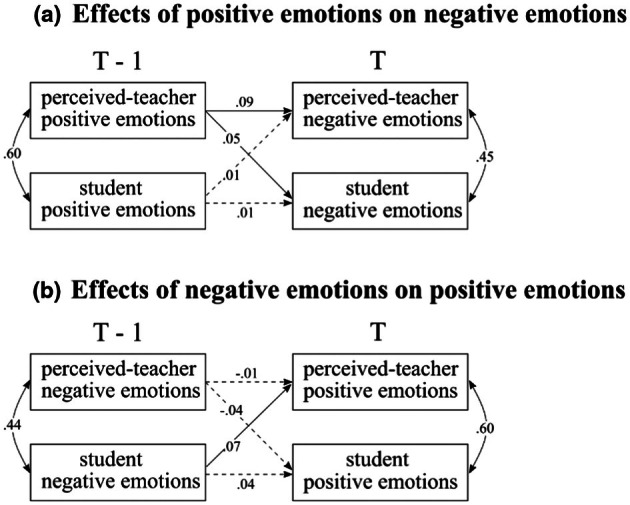
Relationship of cross‐valence transmission between student‐perceived teachers' emotions and students' emotions. Standardized estimates were presented in the figure. Dotted lines represent credible intervals (CI) including zero.

As Figure 6a shows, no effects were found of previous student positive emotions on current student‐perceived teachers' negative emotions (*β* = .01, CI [−.04, .05]) or previous student positive emotions on current student negative emotions (*β* = .01, CI [−.04, .06]). However, student‐perceived teachers' positive emotions in the previous lessons predicted current students' perceptions of teachers' negative emotions (*β* = .09, CI [.04, .14]) and student negative emotions (*β* = .05, CI [.00, .10]). Student‐perceived teachers' positive emotions were correlated with students' positive emotions at each timepoint (previous (T−1): *ρ* = .60, CI [.57, .62]; current (T): *ρ* = .45, CI [.42, .48]).

In terms of previous negative emotions and current positive emotions (Figure 6b), previous students' negative emotions predicted current student‐perceived teachers' positive emotions (*β* = .07, CI [.03, .12]). However, we did not find other cross‐valence effects.

#### Teachers' and students' self‐reported emotions

**FIGURE 7:**
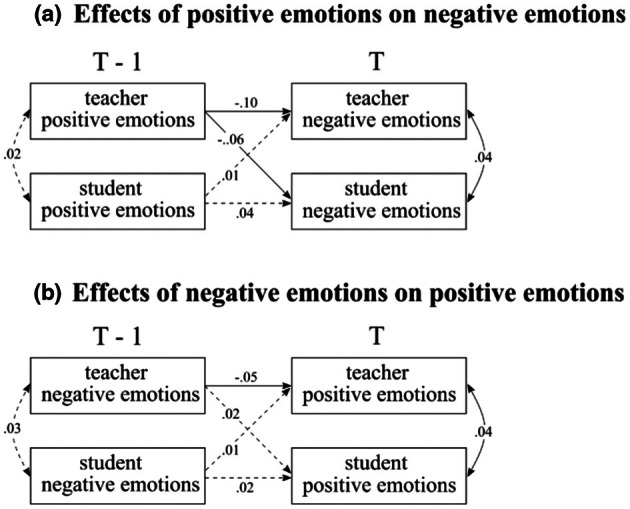
Relationship between cross‐valence emotions as reported by teacher and student. Standardized estimates were presented in the figure. Dotted lines represent credible intervals (C.I.) including zero.

We examined the relationships of cross‐valence emotions between students' self‐reports and teachers' self‐reports. As Figure 7a shows, we found that previous teachers' positive emotions negatively predicted subsequent students' (*β* = −.06, CI [−.10, −.02]) and teachers' negative emotions (*β* = −.10, CI [−.14, −.05]). The higher the teachers' positive emotions in the previous lesson, the more students reported lower negative emotions in subsequent lessons. However, there was no credible effect of previous students' positive emotions on their own subsequent negative emotions (*β* = .04, CI [.00, .08]) or on teachers' subsequent negative emotions (*β* = .01, CI [−.04, .05]).

Figure 7b illustrates the effect of teachers' and students' negative emotions from previous lessons on their positive emotions in subsequent lessons. The analysis found no credible effects of previous negative emotions from either teachers (*β* = .02, CI [−.02, .06]) or students (*β* = .02, CI [−.02, .07]) on the positive emotions of students in subsequent lessons. Additionally, we found no effect of previous students' negative emotions on current teachers' positive emotions (*β* = .01, CI [−.03, .05]). However, previous teachers' negative emotions negatively predicted current teachers' positive emotions (*β* = −.05, CI [−.10, −.01]). This indicates that the higher the teacher‐rated negative emotions in previous lessons, the less they reported experiencing positive emotions in subsequent lessons.

## DISCUSSION

This study contributes to the literature by investigating the reciprocal relationships between situation‐specific emotions of primary school teachers and students, and student‐reported teachers' emotions. Our findings highlight how current emotions are linked to emotions from the previous lesson, incorporating both teachers' and students' experiences through cross‐classified models. In the following sections, we discuss the findings in the order of the research questions and hypotheses.

### Situational variance of student and teacher emotions

Consistent with our first hypothesis we found that most variation in students' emotions and student‐perceived teachers' emotions, specifically negative ones, was between lessons. This is partly in line with the findings of previous experience sampling (Goetz et al., 2013) and cross‐classified research (Mainhard et al., 2018), indicating that considerable variation is situated at the lesson level.

Notably, most previous intra‐individual research on discrete emotions (e.g., enjoyment, anger, anxiety) has focused on two‐level models about student reports and discovered that around 70%–80% of secondary school students' emotions were attributable to between‐situation variations (e.g., Becker et al., 2014; Keller & Becker, 2021). Similar results have been found for student‐perceived teachers' enjoyment, anger and anxiety (59%–81%, Keller & Becker, 2021). However, our findings point out the different patterns of positive and negative emotions among primary school students in Taiwan. Primary school students tended to attribute teachers' negative emotions to situations but viewed teachers' positive emotions as more stable across lessons. This difference in attribution suggests that future research could further explore how students develop their perceptions of teachers' emotions and whether these attributions vary across cultures.

Interestingly, consistent with the findings of Li et al. (2022, 2024), the most variability in teachers' positive and negative emotions was found at the teacher level, suggesting teachers' emotional experiences in this study are relatively less state‐like and more trait‐like. This result may reflect that emotions could be understood as a state–trait continuum (Frenzel et al., 2021). Situational emotions may reflect individuals' connection to their socially constructed beliefs and reactions to various situations (Schutz et al., 2006, p. 344). Moreover, teachers in Taiwan may express a lower intensity of emotions and thus more likely to employ emotional self‐regulation strategies to maintain an organized classroom environment and meet social expectations (Yin & Lee, 2012). For example, teachers might distance themselves from their emotions or suppress them to uphold stability and harmony. These strategies could contribute to the relatively stable trends observed in teachers' emotions in this study.

### Stability from one lesson to the next

Consistent with the second hypothesis, our findings showed that teachers' emotions in a previous lesson predicted their emotions in the current lesson. Similarly, students' emotions also showed some temporal continuity across lessons, though with a smaller effect (*β*: .14–.17; Fey et al., 2023), suggesting changes in emotions across lessons are relatively short‐lived. These findings align with prior research on emotional dynamics in university students (Koval et al., 2016; Kuppens et al., 2010) and extend it to students' perceptions of teachers' emotions in the primary school context. Furthermore, our findings support the broaden‐and‐build theory (Fredrickson, 2001, 2004), showing that both positive and negative emotions can have carryover effects across lessons. This further indicates that emotions in the classroom are not isolated experiences, and that these ongoing experiences may associate with well‐being or adjustment (e.g., Koval et al., 2016).

### Same‐valence emotion transmission between teachers and students

We found that student‐perceived teachers' emotions in the previous lesson predicted both students' emotions and student‐perceived teachers' emotions in subsequent lessons. Previous student‐perceived teachers' positive and negative emotions predicted current students' positive and negative emotions, respectively, with students' previous negative emotions associated with their subsequent perceptions of their teachers' negative emotions. Our findings expand previous studies regarding the concurrent relationships and suggest the same‐valence reciprocal emotion transmission between student‐perceived teachers' and students' emotions. This result implies that students' emotions and their perceptions of teachers' emotions interact reciprocally over time, highlighting the significant role of student appraisals in shaping emotional experiences (Pekrun, 2006). Students' perceptions of teachers' emotions may affect their evaluations of control over and value of learning, which in turn shape their own emotions. These emotions may then influence how students interpret teachers' emotions, thereby reinforcing an emotional feedback loop.

Furthermore, our analysis revealed a delayed but direct positive emotional transmission from teachers' self‐reported positive emotions to students' positive emotions, but not the other way around. To some extent, this finding aligns with previous research that teacher–student emotion transmission over time follows a teacher‐led pattern (Frenzel et al., 2024). Teachers are more likely to transmit their emotions to students, with positive emotions being particularly influential. One possibility is that when a teacher interacts with a group of students displaying diverse emotions, the lack of emotional cohesion may reduce the collective effects of students' emotions on the teacher's emotions. Alternatively, particular students in the class may affect teachers' emotions more strongly. When a teacher was generally more involved with one or more particular students, and these students showed greater engagement in class, the teacher tended to experience a higher level of positive emotions and a lower level of negative emotions (see Li et al., 2024). The small effect of teachers' self‐reported emotions on students' reports may be attributed to teachers self‐regulating their emotions in response to students or to teachers' underreporting their emotions. These factors may complicate the direct pathway between teacher and student emotions. As the findings suggest that only teachers' previous positive emotions predict students' current positive emotions, it is worth noting that a teacher may first interact with one student, transmitting emotion to that student, who then transmits it to others.

### Relationship of cross‐valence emotions between teachers and students

Although the concept of cross‐valence emotion transmission has been discussed in other interpersonal contexts such as parent and adolescent (e.g., Mancini et al., 2016), it has not been widely studied in the classroom setting. This study initiates the exploration by examining what is the relationship of cross‐valence (e.g., positive to negative) emotions using both teachers' and students' reports. Overall, we found that teachers' higher positive emotions in previous lessons protected both teachers and students from experiencing higher levels of negative emotions later.

Interestingly, we found a weak but credible positive relationship between students' negative emotions in the previous lesson and their perceptions of teachers' positive emotions in the current lesson. This finding suggests that when students experienced stronger negative emotions, teachers may have responded by showing more positive emotions to alleviate students' distress and foster a more positive emotional climate (Jia & Cheng, 2024). However, an unanticipated finding was that although teachers' positive emotions, as perceived by students in a previous lesson, predicted students' positive emotions, it also weakly but positively predicted both students' perceptions of teachers' negative emotions and students' negative emotions in the current lesson. This result echoes the findings of Hamaker et al. (2018), who found that positive emotions had a weak but significant positive association with negative emotions the next day, which they did not find in the between‐person correlations. This may be because individuals with a tendency to experience positive emotions intensely also tend to experience negative emotions more intensely. In our case, students who tend to perceive teachers as having higher positive emotions also tend to perceive teachers as having higher negative emotions across lessons. Another possible explanation is that teachers may display mixed emotional signals during a lesson, which primary school students might interpret as unfriendly cues, leading to subsequent negative emotions (Gillis & Nilsen, 2017). Additionally, students might perceive a teacher's emotional expression as unauthentic, potentially intensifying their own negative emotions (Keller & Becker, 2021).

Taken together, the findings on student‐perceived teachers' emotions and student emotions reflect the emotional complexity inherent in classroom dynamics, where positive and negative emotions often coexist rather than being strictly bipolar (Dejonckheere et al., 2019). Future research is needed to further explore these complex cross‐valence emotional dynamics.

## LIMITATIONS

One limitation of this study relates to the sample of participants. Participation in this study was voluntary, suggesting that this could lead to self‐selection bias in the study, if certain participants were more willing to join the study than others. For example, teachers who were more motivated or confident about their teaching may have been more likely to participate. Moreover, the sample size of teacher participants was relatively small. In this study, emotion transmission was analysed using time points (lessons), with priors tailored to our relatively small sample of 20 teachers (McNeish, 2019). Future research would benefit from validating the findings with a larger sample of teachers.

Secondly, although this study included both student and teacher reports to minimize self‐report bias and investigated the transmission mechanism between teachers and students, it should be noted that this study, still, is based on self‐reported data. Integrating other measures (e.g., observations) would be of benefit in future research.

Furthermore, we did not include teacher‐perceived students' emotions in this study and therefore could not conduct a parallel analysis to investigate whether teacher‐perceived students' emotions correspond with students' emotions. As we found that students cannot always precisely identify teachers' emotions in the classroom, it would be interesting to explore whether this is the case for teachers. Additionally, we grouped teachers' and students' discrete emotions into positive and negative emotions to investigate emotional transmission in school settings. The relationships between teachers' and students' specific discrete emotions (e.g., anxiety, anger, enjoyment, pride) are worth further discussion.

Finally, we did not investigate what specific events teachers or students experienced in the lesson that might have elicited their positive and negative emotions or even a certain discrete emotion (e.g., a teacher's anger arose because a student chatted loudly). Future studies could consider linking specific antecedents and emotions to deepen our understanding of the emotional transmission mechanism.

## IMPLICATIONS AND CONCLUSIONS

In summary, this study provides insights into emotion dynamics by examining the associations between homeroom teachers' emotions and students within the context of their shared experiences. It broadens our understanding of emotion transmission between teachers and students by incorporating a mid‐range timescale (i.e., lesson‐level) grounded in actual instructional episodes and incorporating both sides of the teacher–student interaction.

Our study supports that students' perceptions of teachers' positive emotions can serve as a source to elicit students' positive emotions, thereby contributing to a positive learning environment. This finding underlines the importance of students' perceptions of their teachers' emotions in shaping their own emotional experiences. Additionally, like students' perceptions of teachers' positive emotions, we found that teachers' own positive emotions affect students' subsequent positive emotions. However, students' positive emotions did not have a comparable effect on teachers. This finding supports the idea that teachers are more likely to be the senders in the emotion transmission process. According to emotional contagion theory, this may be attributed to the role of teachers, which involves greater control and authority in the classroom (Hatfield et al., 1994).

In addition, teachers' elevated positive emotions may dampen students' negative emotional experience, although students may still carry their negative emotions over. These results suggest that interventions aiming to enhance classroom emotional experiences should target teachers and students simultaneously. For teachers, interventions could focus on increasing their positive emotional experiences over time, thereby fostering a more positive classroom climate. For students, supporting students to recognize their negative emotions and gain positive emotional experiences may help them decrease the accumulation of negative emotions and build personal resources.

In classroom settings, it is common for teachers to display their emotions following certain implicit rules in specific cultural contexts. Interestingly, Taiwanese culture, which addresses interpersonal harmony, could encourage emotional suppression to find the appropriate response and to preserve relationships, which might be used on both negative and positive emotions (Hosotani & Imai‐Matsumura, 2011; Matsumoto et al., 2008; Yeh et al., 2017). Although it may have a prosocial function, it could decrease the emotional authenticity of teachers' emotions (Keller & Becker, 2021). An encouraging finding of this study is the weak effect of teachers' previous positive emotions on students' higher positive and lower negative emotions. Sharing emotional experiences rather than suppressing negative emotions or pretending to have positive emotions might be more beneficial to teachers and students. By sharing emotional experiences collectively, teachers and students express emotions and explain what might cause their emotions, improving their understanding of each other and their positive emotions.

In conclusion, our findings support the emotional transmission between student‐perceived teachers' emotions and students' emotions across lessons. Further, the reciprocal relationship between teacher and student emotions indicated that while teachers' emotions are mainly based on their own prior emotions, students' emotions were predicted by perceived teachers' and students' prior emotions. By examining lagged effects, our study provides a clearer picture of teacher–student emotion transmission, highlighting the crucial role of teachers' emotions in the reciprocal relationships between teachers' and students' emotions.

## AUTHOR CONTRIBUTIONS

**Pei‐Hsin Li:** Conceptualization; methodology; investigation; formal analysis; project administration; writing – original draft; writing – review and editing. **Diane Mayer:** Supervision; writing – review and editing. **Lars‐Erik Malmberg:** Supervision; writing – review and editing; conceptualization; validation; formal analysis.

## CONFLICT OF INTEREST STATEMENT

None.

## Supporting information


Appendix S1

## Data Availability

Research data supporting the results is shared for the peer review process.

## Footnotes

^1^
Lagged variables were generated using data from lessons conducted each day. Due to the extended time intervals between days, the lagged values of the first lesson for each day were coded missing. To create meaningful lagged variables, missing data points (*n* = 189) were inserted.

^2^
A further analysis was conducted to explore if students' perceptions of their teachers' emotions would mediate the association between teachers' and students' emotions. As shown in the Appendix S1, we found no mediation over time. However, at the concurrent timepoint, indirect effects were found for both positive and negative emotions.

## APPENDIX A PARTICIPATION RATE IN EACH CLASS

**Table:**

Class	Students (total)	Participation rate (class)
1	22 (28)	.79
2	17 (27)	.63
3	21 (27)	.78
4	22 (26)	.85
5	12 (28)	.43
6	6 (25)	.24
7	19 (23)	.83
8	11 (24)	.46
9	13 (24)	.54
10	20 (23)	.87
11	12 (25)	.48
12	15 (24)	.63
13	8 (24)	.33
14	20 (24)	.83
15	17 (25)	.68
16	11 (26)	.42
17	11 (21)	.52
18	12 (20)	.60
19	21 (21)	1.00
20	18 (20)	.90
	308 (485)	

## References

[bjep70006-bib-0001] Ariens, S. , Ceulemans, E. , & Adolf, J. K. (2020). Time series analysis of intensive longitudinal data in psychosomatic research: A methodological overview. Journal of Psychosomatic Research, 137, 110191. 10.1016/j.jpsychores.2020.110191 32739633

[bjep70006-bib-0002] Asparouhov, T. , & Muthén, B. (2020). Comparison of models for the analysis of intensive longitudinal data. Structural Equation Modeling: A Multidisciplinary Journal, 27(2), 275–297. 10.1080/10705511.2019.1626733

[bjep70006-bib-0003] Becker, E. S. , Goetz, T. , Morger, V. , & Ranellucci, J. (2014). The importance of teachers' emotions and instructional behavior for their students' emotions: An experience sampling analysis. Teaching and Teacher Education, 43, 15–26. 10.1016/j.tate.2014.05.002

[bjep70006-bib-0004] Becker, E. S. , Keller, M. M. , Goetz, T. , Frenzel, A. C. , & Taxer, J. L. (2015). Antecedents of teachers' emotions in the classroom: An intraindividual approach. Frontiers in Psychology, 6, 635. 10.3389/fpsyg.2015.00635 26042067 PMC4436560

[bjep70006-bib-0005] Beretvas, S. N. (2011). Cross‐classified and multiple‐membership models. In J. Hox & J. K. Roberts (Eds.), Handbook of advanced multilevel analysis. Taylor & Francis Group. http://ebookcentral.proquest.com/lib/oxford/detail.action?docID=668990

[bjep70006-bib-0007] Bolger, N. , & Laurenceau, J.‐P. (2013). Intensive longitudinal methods: An introduction to diary and experience sampling research (p. xv, 256). Guilford Press.

[bjep70006-bib-0006] Bolger, N. , DeLongis, A. , Kessler, R. C. , & Wethington, E. (1989). The contagion of stress across multiple roles. Journal of Marriage and the Family, 51(1), 175. 10.2307/352378

[bjep70006-bib-0008] Butler, E. A. (2011). Temporal interpersonal emotion systems: The “TIES” that form relationships. Personality and Social Psychology Review, 15(4), 367–393. 10.1177/1088868311411164 21693670

[bjep70006-bib-0009] Camacho‐Morles, J. , Slemp, G. R. , Pekrun, R. , Loderer, K. , Hou, H. , & Oades, L. G. (2021). Activity achievement emotions and academic performance: A meta‐analysis. Educational Psychology Review, 33(3), 1051–1095. 10.1007/s10648-020-09585-3

[bjep70006-bib-0010] Chang, Y.‐F. , & Cherng, B.‐L. (2017). The relations of teachers' teaching emotions, students' achievement emotions, and students' motivational engagement for junior high school students. Bulletin of Educational Psychology, 49(1), 113–136. 10.6251/BEP.20161028

[bjep70006-bib-0011] Crawford, J. R. , & Henry, J. D. (2004). The positive and negative affect schedule (PANAS): Construct validity, measurement properties and normative data in a large non‐clinical sample. British Journal of Clinical Psychology, 43(3), 245–265. 10.1348/0144665031752934 15333231

[bjep70006-bib-0012] de Ruiter, J. A. , Poorthuis, A. M. G. , & xaaaa, H. M. Y. (2021). Teachers' emotional labor in response to daily events with individual students: The role of teacher–student relationship quality. Teaching and Teacher Education, 107, 103467. 10.1016/j.tate.2021.103467

[bjep70006-bib-0013] Dejonckheere, E. , Kalokerinos, E. K. , Bastian, B. , & Kuppens, P. (2019). Poor emotion regulation ability mediates the link between depressive symptoms and affective bipolarity. Cognition & Emotion, 33(5), 1076–1083. 10.1080/02699931.2018.1524747 30270738

[bjep70006-bib-0014] Delvaux, E. , Meeussen, L. , & Mesquita, B. (2016). Emotions are not always contagious: Longitudinal spreading of self‐pride and group pride in homogeneous and status‐differentiated groups. Cognition and Emotion, 30(1), 101–116. 10.1080/02699931.2015.1018143 25786806

[bjep70006-bib-0015] Fey, C. F. , Hu, T. , & Delios, A. (2023). The measurement and communication of effect sizes in management research. Management and Organization Review, 19(1), 176–197. 10.1017/mor.2022.2

[bjep70006-bib-0016] Fielding, A. , & Goldstein, H. (2006). Cross‐classified and multiple membership structures in multilevel models: (Research report RR791) (pp. 1–69). University of Birmingham.

[bjep70006-bib-0017] Fredrickson, B. L. (2001). The role of positive emotions in positive psychology. American Psychologist, 56(3), 218–226. 11315248 10.1037//0003-066x.56.3.218 PMC3122271

[bjep70006-bib-0018] Fredrickson, B. L. (2004). The broaden‐and‐build theory of positive emotions. Philosophical Transactions of the Royal Society of London. Series B, Biological Sciences, 359(1449), 1367–1378. 10.1098/rstb.2004.1512 15347528 PMC1693418

[bjep70006-bib-0019] Frenzel, A. C. (2014). Teacher emotions. In E. A. Linnenbrink‐Garcia , R. Pekrun , E. A. Linnenbrink‐Garcia , & R. Pekrun (Eds.), International handbook of emotions in education (pp. 494–519). Routledge.

[bjep70006-bib-0020] Frenzel, A. C. , Becker‐Kurz, B. , Pekrun, R. , & Goetz, T. (2015). Teaching this class drives me nuts! – Examining the person and context specificity of teacher emotions. PLoS One, 10(6), e0129630. 10.1371/journal.pone.0129630 26053623 PMC4459880

[bjep70006-bib-0021] Frenzel, A. C. , Becker‐Kurz, B. , Pekrun, R. , Goetz, T. , & Lüdtke, O. (2018). Emotion transmission in the classroom revisited: A reciprocal effects model of teacher and student enjoyment. Journal of Educational Psychology, 110(5), 628–639. 10.1037/edu0000228

[bjep70006-bib-0022] Frenzel, A. C. , Daniels, L. , & Burić, I. (2021). Teacher emotions in the classroom and their implications for students. Educational Psychologist, 56(4), 250–264. 10.1080/00461520.2021.1985501

[bjep70006-bib-0023] Frenzel, A. C. , Dindar, M. , Pekrun, R. , Reck, C. , & Marx, A. K. G. (2024). Joy is reciprocally transmitted between teachers and students: Evidence on facial mimicry in the classroom. Learning and Instruction, 91, 101896. 10.1016/j.learninstruc.2024.101896

[bjep70006-bib-0024] Frenzel, A. C. , Goetz, T. , Stephens, E. J. , & Jacob, B. (2009). Antecedents and effects of teachers' emotional experiences: An integrated perspective and empirical test. In P. A. Schutz & M. Zembylas (Eds.), Advances in teacher emotion research (pp. 129–151). Springer US. 10.1007/978-1-4419-0564-2_7

[bjep70006-bib-0025] Gillis, R. L. , & Nilsen, E. S. (2017). Consistency between verbal and non‐verbal affective cues: A clue to speaker credibility. Cognition and Emotion, 31(4), 645–656. 10.1080/02699931.2016.1147422 26892724

[bjep70006-bib-0026] Glomb, T. M. , & Tews, M. J. (2004). Emotional labor: A conceptualization and scale development. Journal of Vocational Behavior, 64(1), 1–23. 10.1016/S0001-8791(03)00038-1

[bjep70006-bib-0027] Goetz, T. , Becker, E. S. , Bieg, M. , Keller, M. M. , Frenzel, A. C. , & Hall, N. C. (2015). The glass half empty: How emotional exhaustion affects the state‐trait discrepancy in self‐reports of teaching emotions. PLoS One, 10(9), e0137441. 10.1371/journal.pone.0137441 26368911 PMC4569532

[bjep70006-bib-0028] Goetz, T. , Keller, M. M. , Lüdtke, O. , Nett, U. E. , & Lipnevich, A. A. (2020). The dynamics of real‐time classroom emotions: Appraisals mediate the relation between students' perceptions of teaching and their emotions. Journal of Educational Psychology, 112(6), 1243–1260.

[bjep70006-bib-0029] Goetz, T. , Lüdtke, O. , Nett, U. E. , Keller, M. M. , & Lipnevich, A. A. (2013). Characteristics of teaching and students' emotions in the classroom: Investigating differences across domains. Contemporary Educational Psychology, 38(4), 383–394. 10.1016/j.cedpsych.2013.08.001

[bjep70006-bib-0030] Gurtman, M. B. , & Pincus, A. L. (2003). The circumplex model: Methods and research applications. In J. A. Schinka & W. F. Velicer (Eds.), Handbook of psychology: Research methods in psychology (Vol. 2, pp. 407–428). John Wiley & Sons, Inc. 10.1002/0471264385.wei0216

[bjep70006-bib-0031] Hagenauer, G. , Hascher, T. , & Volet, S. E. (2015). Teacher emotions in the classroom: Associations with students' engagement, classroom discipline and the interpersonal teacher‐student relationship. European Journal of Psychology of Education, 30(4), 385–403. 10.1007/s10212-015-0250-0

[bjep70006-bib-0032] Hamaker, E. L. , Asparouhov, T. , Brose, A. , Schmiedek, F. , & Muthén, B. (2018). At the frontiers of modeling intensive longitudinal data: Dynamic structural equation models for the affective measurements from the COGITO study. Multivariate Behavioral Research, 53(6), 820–841. 10.1080/00273171.2018.1446819 29624092

[bjep70006-bib-0033] Hargreaves, A. (2000). Mixed emotions: Teachers' perceptions of their interactions with students. Teaching and Teacher Education, 16(8), 811–826. 10.1016/S0742-051X(00)00028-7

[bjep70006-bib-0034] Harmon‐Jones, E. , Harmon‐Jones, C. , & Summerell, E. (2017). On the importance of both dimensional and discrete models of emotion. Behavioral Science, 7(4), 66. 10.3390/bs7040066 PMC5746675 28961185

[bjep70006-bib-0035] Hatfield, E. , Cacioppo, J. T. , & Rapson, R. L. (1994). Emotional contagion (1st ed.). Cambridge University Press.

[bjep70006-bib-0073] Herrando, C. , & Constantinides, E. (2021). Emotional contagion: A brief overview and future directions. Frontiers in Psychology, 12. 10.3389/fpsyg.2021.712606 PMC8322226 34335425

[bjep70006-bib-0036] Hosotani, R. , & Imai‐Matsumura, K. (2011). Emotional experience, expression, and regulation of high‐quality Japanese elementary school teachers. Teaching and Teacher Education, 27(6), 1039–1048. 10.1016/j.tate.2011.03.010

[bjep70006-bib-0037] Hox, J. , van de Schoot, R. , & Matthijsse, S. (2012). How few countries will do? Comparative survey analysis from a Bayesian perspective. Survey Research Methods, 6(2), 2. 10.18148/srm/2012.v6i2.5033

[bjep70006-bib-0038] Hubbard, J. A. , Moore, C. C. , Zajac, L. , Bookhout, M. K. , & Dozier, M. (2023). Emotion transmission in peer dyads in middle childhood. Child Development, 94(4), 1017–1032. 10.1111/cdev.13917 36892485

[bjep70006-bib-0039] Jia, M. , & Cheng, J. (2024). Effect of teacher social support on students' emotions and learning engagement: A U.S.‐Chinese classroom investigation. Humanities and Social Sciences Communications, 11(1), 158. 10.1057/s41599-024-02634-0

[bjep70006-bib-0040] Keller, M. M. , & Becker, E. S. (2021). Teachers' emotions and emotional authenticity: Do they matter to students' emotional responses in the classroom? Teachers and Teaching, 27(5), 404–422. 10.1080/13540602.2020.1834380

[bjep70006-bib-0074] Keller, M. M. , Yanagida, T. , Lüdtke, O. , & Goetz, T. (2025). How similar are students’ aggregated state emotions to their self‐reported trait emotions? Results from a measurement burst design across three school years. Educational Psychology Review, 37(1). 10.1007/s10648-025-09995-1 PMC11906532 40092058

[bjep70006-bib-0041] Koval, P. , Sütterlin, S. , & Kuppens, P. (2016). Emotional inertia is associated with lower well‐being when controlling for differences in emotional context. Frontiers in Psychology, 6, 1997. 10.3389/fpsyg.2015.01997 26779099 PMC4705270

[bjep70006-bib-0042] Kuppens, P. , Allen, N. B. , & Sheeber, L. (2010). Emotional inertia and psychological maladjustment. Psychological Science, 21(7), 984–991. 10.1177/0956797610372634 20501521 PMC2901421

[bjep70006-bib-0043] Larson, R. W. , & Almeida, D. M. (1999). Emotional transmission in the daily lives of families: A new paradigm for studying family process. Journal of Marriage and Family, 61(1), 5–20. 10.2307/353879

[bjep70006-bib-0044] Li, P. H. , Mayer, D. , & Malmberg, L.‐E. (2022). Teacher well‐being in the classroom: A micro‐longitudinal study. Teaching and Teacher Education, 115, 103720. 10.1016/j.tate.2022.103720

[bjep70006-bib-0045] Li, P. H. , Mayer, D. , & Malmberg, L.‐E. (2024). Student engagement and teacher emotions in student‐teacher dyads: The role of teacher involvement. Learning and Instruction, 91, 101876. 10.1016/j.learninstruc.2024.101876

[bjep70006-bib-0046] Lin, D. , Zhu, T. , & Wang, Y. (2024). Emotion contagion and physiological synchrony: The more intimate relationships, the more contagion of positive emotions. Physiology & Behavior, 275, 114434. 10.1016/j.physbeh.2023.114434 38092069

[bjep70006-bib-0047] Mainhard, T. , Oudman, S. , Hornstra, L. , Bosker, R. J. , & Goetz, T. (2018). Student emotions in class: The relative importance of teachers and their interpersonal relations with students. Learning and Instruction, 53, 109–119. 10.1016/j.learninstruc.2017.07.011

[bjep70006-bib-0048] Malmberg, L.‐E. , & Martin, A. J. (2019). Processes of students' effort exertion, competence beliefs and motivation: Cyclic and dynamic effects of learning experiences within school days and school subjects. Contemporary Educational Psychology, 58, 299–309. 10.1016/j.cedpsych.2019.03.013

[bjep70006-bib-0049] Mancini, K. J. , Luebbe, A. M. , & Bell, D. J. (2016). Valence‐specific emotion transmission: Potential influences on parent–adolescent emotion coregulation. Emotion, 16(5), 567–574. 10.1037/emo0000160 26986488

[bjep70006-bib-0050] Matsumoto, D. , Yoo, S. H. , & Nakagawa, S. (2008). Culture, emotion regulation, and adjustment. Journal of Personality and Social Psychology, 94(6), 925. 10.1037/0022-3514.94.6.925 18505309

[bjep70006-bib-0052] McNeish, D. (2019). Two‐level dynamic structural equation models with small samples. Structural Equation Modeling: A Multidisciplinary Journal, 26(6), 948–966. 10.1080/10705511.2019.1578657 32863699 PMC7451754

[bjep70006-bib-0053] Mottet, T. , & Beebe, S. (2000). Emotional contagion in the classroom: An examination of how teacher and student emotions are related. Annual Meeting of the National Communication Association.

[bjep70006-bib-0054] Muthén, L. K. , & Muthén, B. O. (1998–2017). Mplus user's guide. [Computer software]. Muthén & Muthén.

[bjep70006-bib-0055] Olszanowski, M. , Wrobel, M. , & Hess, U. (2019). Mimicking and sharing emotions: A re‐examination of the link between facial mimicry and emotional contagion. Cognition and Emotion, 34(2), 367–376. 10.1080/02699931.2019.1611543 31072246

[bjep70006-bib-0056] Pekrun, R. (2006). The control‐value theory of achievement emotions: Assumptions, corollaries, and implications for educational research and practice. Educational Psychology Review, 18(4), 315–341. 10.1007/s10648-006-9029-9

[bjep70006-bib-0057] Pekrun, R. (2024). Control‐value theory: From achievement emotion to a general theory of human emotions. Educational Psychology Review, 36(3), 83. 10.1007/s10648-024-09909-7

[bjep70006-bib-0058] Pekrun, R. , Goetz, T. , Titz, W. , & Perry, R. P. (2002). Academic emotions in students' self‐regulated learning and achievement: A program of qualitative and quantitative research. Educational Psychologist, 37(2), 91–105. 10.1207/S15326985EP3702_4

[bjep70006-bib-0059] Pekrun, R. , Lichtenfeld, S. , Marsh, H. W. , Murayama, K. , & Goetz, T. (2017). Achievement emotions and academic performance: Longitudinal models of reciprocal effects. Child Development, 88(5), 1653–1670. 10.1111/cdev.12704 28176309

[bjep70006-bib-0060] Pons, F. , Harris, P. L. , & de Rosnay, M. (2004). Emotion comprehension between 3 and 11 years: Developmental periods and hierarchical organization. European Journal of Developmental Psychology, 1(2), 127–152. 10.1080/17405620344000022

[bjep70006-bib-0061] Prosen, S. , Vitulić, H. , & Škraban, O. (2011). Teachers' emotional expression in interaction with students of different ages. Center for Educational Policy Studies Journal, 1, 141–157. 10.26529/cepsj.419

[bjep70006-bib-0062] Sanders, E. A. , & Konold, T. R. (2023). X matters too: How the blended slope problem manifests differently in unilevel vs. multilevel models. Methodology, 19(1), 1–23. 10.5964/meth.9925

[bjep70006-bib-0063] Schutz, P. A. (2014). Inquiry on teachers' emotion. Educational Psychologist, 49(1), 1–12. 10.1080/00461520.2013.864955

[bjep70006-bib-0064] Schutz, P. A. , Hong, J. Y. , Cross, D. I. , & Osbon, J. N. (2006). Reflections on investigating emotion in educational activity settings. Educational Psychology Review, 18(4), 343–360. 10.1007/s10648-006-9030-3

[bjep70006-bib-0065] Tam, K. Y. Y. , Poon, C. Y. S. , Hui, V. K. Y. , Wong, C. Y. F. , Kwong, V. W. Y. , Yuen, G. W. C. , & Chan, C. S. (2019). Boredom begets boredom: An experience sampling study on the impact of teacher boredom on student boredom and motivation. British Journal of Educational Psychology, 90(S1), 124–137. 10.1111/bjep.12309 31342514

[bjep70006-bib-0066] Trampe, D. , Quoidbach, J. , & Taquet, M. (2015). Emotions in everyday life. PLoS One, 10(12), e0145450. 10.1371/journal.pone.0145450 26698124 PMC4689475

[bjep70006-bib-0067] Watson, D. , & Tellegen, A. (1985). Toward a consensual structure of mood. Psychological Bulletin, 98(2), 219–235. 10.1037/0033-2909.98.2.219 3901060

[bjep70006-bib-0068] Westman, M. , Shadach, E. , & Keinan, G. (2013). The crossover of positive and negative emotions: The role of state empathy. International Journal of Stress Management, 20(2), 116–133. 10.1037/a0033205

[bjep70006-bib-0069] Yeh, K.‐H. , Bedford, O. , Wu, C. , Wang, S.‐Y. , & Yen, N.‐S. (2017). Suppression benefits boys in Taiwan: The relation between gender, emotional regulation strategy, and mental health. Frontiers in Psychology, 8, 135. 10.3389/fpsyg.2017.00135 28220099 PMC5292407

[bjep70006-bib-0070] Yin, H. , & Lee, J. C.‐K. (2012). Be passionate, but be rational as well: Emotional rules for Chinese teachers' work. Teaching and Teacher Education, 28(1), 56–65. 10.1016/j.tate.2011.08.005

