# Supplementary material for: Are teachers' and students' emotions reciprocally transmitted in the classroom?
Source: Br J Educ Psychol. 2025 Jun 27;95(Suppl 1):S172–93. doi: 10.1111/bjep.70006 (PMC12427168; doi:10.1111/bjep.70006)
Supplement: Supplementary file 1 — Appendix S1 [file BJEP-95-S172-s001.pdf]

Appendix S1 for ”Are teachers’ and students’ emotions reciprocally transmitted in the classroom?” British Journal of Educational Psychology,

doi = 10.1111/bjep.70006

Version 23 June 2025

Pei-Hsin Li[1,2], Diane Mayer[1], & Lars-Erik Malmberg[1]
[1]University of Oxford, UK; [2]University of Helsinki, Finland

Appendix S1 for ”Are teachers’ and students’ emotions reciprocally transmitted in the classroom?”

Introduction

In these Supplementary Materials we, in more detail, present (1) confirmatory factor analyses of the emotion scales, (2) present alternative auto-regressive models for discrete emotions, (3) give an example of a two-level model in which we specified latent lagged variables and adjusted for non-equidistant time-lags, and (4) analyse whether students’ perceptions of their teacher’s emotions mediated the relationship between teachers’ emotions and students’ own emotions. We are thankful to our reviewers who pointed out the need for these additional analyses.

1. Factor structure

In this section we present cross-classified confirmatory factor analyses (CCCFA). We specified an *a priori* two-factor emotion model for both students and teachers, and contrasted the model fit of these models with those of one-factor models. We observe the factor loadings at the within-level, between-timepoint level, and between-student level, as shown in Fig 1. The two-factor model was specified so that we specified priors for target factor-loadings (λs) at 0.70 with a variance of 0.0, giving $\sqrt{0.04} = \pm\ 0.20$, [0.50,0.90] and the cross-loadings at 0.0 with a variance of 0.04, giving $\sqrt{0.04} = \pm\ 0.20$, [-0.20,0.20](see Muthén & Asparouhov, 2012). The cross-loadings are indicated in grey arrows in Fig 1.

For the two-factor model for the students, the Post-Predictive P-value (PPP) indicated poor model fit, while the 95% confidence interval for the difference between the replicated and observed χ^2 statistics (PPPP) indicated good fit (0.495). Using the Bayesian Factor to compare the one-factor and two-factor models (DIC = 81,886.654 vs. 79,074.813), the two-factor model fitted the data better (BF > 100). When we inspected the factor loadings in Table 1, for positive and negative emotions at the within level we see some cross-loadings (e.g., enjoyment), however the loadings on the target factor were stronger than the loadings on the non-target factor. Cross-loadings at the timepoint level were relatively smaller than those at the within level. There were some cross-loadings at the student level too. Overall, the model gave support for a two-factor solution.

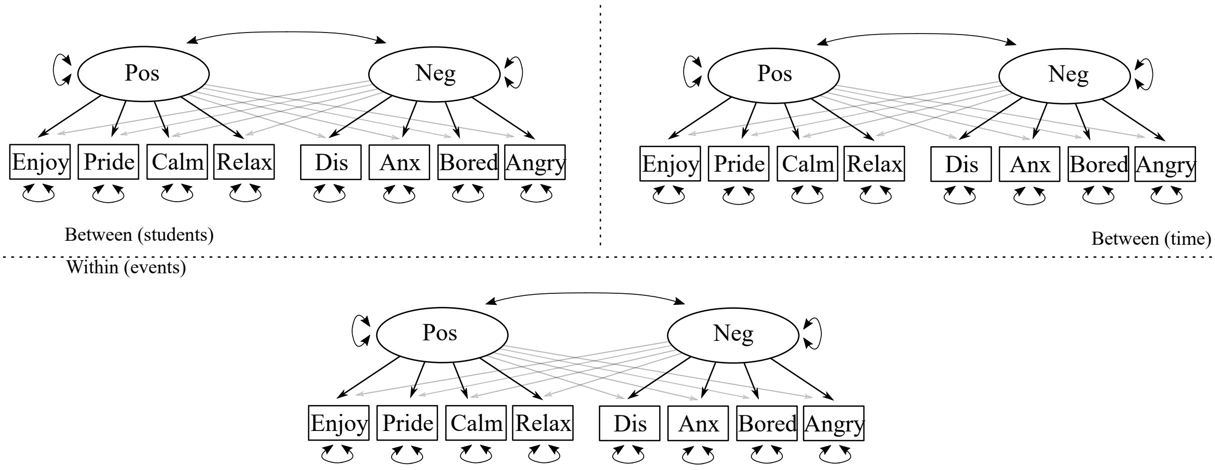

Figure 1. Cross-classified confirmatory factor analysis of positive and negative emotions (two-factor solution), applied to students' and teachers' emotions, at the within-level, between-timepoint level and between-student level.

For the two-factor model for the teachers, the Post-Predictive P-value (PPP) indicated a poor model fit (0.020), while the 95% confidence interval for the difference between the replicated and observed χ^2 statistics (PPPP) indicated good fit (0.348). Using the Bayesian Factor to compare the one-factor and two factor models (DIC = 3609.916 vs 3662.941), the two-factor model fitted the data better (BF > 100), also providing support for the two-factor teacher model. Posterior estimates are provided in Table 2. Our model is different from the random factor slope models suggested by McNeish, Mackinnon, Marsch, and Poldrack (2021). Their model explicitly partitions the variance of the indicators into factor loadings (i.e., common variance, h^2), and uniquenesses (i.e., u^2), at the within, between-timepoints, and between-person levels .

Table 1
Standardised factor loadings with 90% credibility intervals for students' positive and negative emotions, across levels (within, between timepoints, and between students).

Emotion	Pos Emo λ	[90% CI]	Neg emo λ	[90% CI]
Within Level				
Enjoy	0.544	[0.497, 0.591]	-0.272	[-0.359, -0.187]
Pride	0.368	[0.334, 0.406]	0.086	[0.024, 0.148]
Calm	0.422	[0.390, 0.456]	0.073	[0.006, 0.143]
Relaxed	0.742	[0.704, 0.779]	-0.003	[-0.114, 0.110]
Anxious	-0.163	[-0.247, -0.081]	0.488	[0.452, 0.523]
Bored	-0.142	[-0.257, -0.030]	0.701	[0.662, 0.737]
Disappointed	-0.063	[-0.141, 0.008]	0.445	[0.413, 0.479]
Angry	-0.071	[-0.168, 0.029]	0.597	[0.565, 0.631]
Between Level (Timepoints)				
Enjoy	0.804	[0.472, 1.081]	0.010	[-0.229, 0.264]
Pride	0.738	[0.323, 1.097]	0.027	[-0.286, 0.373]
Calm	0.641	[0.252, 0.988]	-0.022	[-0.264, 0.188]
Relaxed	0.575	[0.215, 0.933]	-0.016	[-0.239, 0.184]
Anxious	0.008	[-0.276, 0.270]	0.595	[0.245, 0.943]
Bored	0.025	[-0.454, 0.529]	0.751	[0.268, 1.164]
Disappointed	0.040	[-0.288, 0.430]	0.466	[0.120, 0.853]
Angry	-0.073	[-0.462, 0.213]	0.497	[0.157, 0.846]
Between Level (Students)				
Enjoy	0.734	[0.663, 0.804]	-0.199	[-0.327, -0.067]
Pride	0.519	[0.423, 0.607]	0.127	[0.025, 0.231]
Calm	0.634	[0.541, 0.727]	0.008	[-0.113, 0.124]
Relaxed	0.788	[0.697, 0.883]	-0.001	[-0.136, 0.124]
Anxious	-0.081	[-0.248, 0.108]	0.829	[0.772, 0.884]
Bored	-0.147	[-0.332, 0.061]	0.962	[0.904, 1.010]
Disappointed	-0.187	[-0.323, -0.054]	0.579	[0.495, 0.657]
Angry	-0.084	[-0.256, 0.117]	0.874	[0.818, 0.927]

Table 2

Standardised factor loadings with 90% credibility intervals for teachers' positive and negative emotions, across levels (within, between timepoints, and between teachers.

Emotion	Pos Emo λ	[90% CI]	Neg emo λ	[90% CI]
Within Level				
Enjoy	0.789	[0.641, 0.950]	0.035	[-0.163, 0.227]
Pride	0.595	[0.435, 0.749]	0.152	[-0.026, 0.329]
Calm	0.366	[0.226, 0.509]	-0.077	[-0.231, 0.075]
Relaxed	0.471	[0.342, 0.601]	-0.046	[-0.193, 0.096]
Anxious	0.098	[-0.069, 0.244]	0.674	[0.552, 0.795]
Bored	-0.104	[-0.248, 0.046]	0.577	[0.447, 0.709]
Disappointed	0.045	[-0.122, 0.212]	0.479	[0.331, 0.631]
Angry	-0.072	[-0.228, 0.078]	0.638	[0.502, 0.771]
Between Level (Timepoints)				
Enjoy	0.728	[0.311, 1.058]	-0.016	[-0.409, 0.372]
Pride	0.536	[0.156, 0.954]	0.050	[-0.359, 0.566]
Calm	0.388	[0.069, 0.753]	0.030	[-0.293, 0.391]
Relaxed	0.502	[0.110, 0.914]	-0.041	[-0.480, 0.333]
Anxious	0.010	[-0.193, 0.231]	0.829	[0.472, 1.052]
Bored	-0.015	[-0.302, 0.197]	0.646	[0.269, 1.000]
Disappointed	0.008	[-0.263, 0.319]	0.768	[0.355, 1.046]
Angry	-0.003	[-0.280, 0.255]	0.747	[0.323, 1.038]
Between Level (Teachers)				
Enjoy	0.995	[0.755, 1.255]	0.048	[-0.254, 0.342]
Pride	0.835	[0.541, 1.105]	-0.030	[-0.336, 0.262]
Calm	0.701	[0.401, 0.976]	-0.126	[-0.460, 0.195]
Relaxed	0.604	[0.326, 0.901]	0.029	[-0.236, 0.319]
Anxious	0.072	[-0.123, 0.302]	0.756	[0.540, 0.986]
Bored	-0.009	[-0.284, 0.250]	0.953	[0.706, 1.173]
Disappointed	-0.202	[-0.459, 0.025]	0.766	[0.545, 0.973]
Angry	0.028	[-0.258, 0.308]	0.934	[0.649, 1.174]

2. Discrete emotions

We inspected students' discrete emotions by specifying cross-classified auto-regressive models for each discrete emotion regressed on its lagged emotion at the within-level, and estimated the variance of that emotion across students, and across teachers. We present only the within-part of the models in Fig 2. All discrete emotions were somewhat stable across time, with unstandardised coefficients between B = 0.059 to 0.134 and standardised coefficients between $\beta = 0.055$ to 0.124. As the CCCFA fitted data well as shown in the previous section, we found the scale-scores for positive and negative emotions appropriate for modelling rather than the discrete emotions.

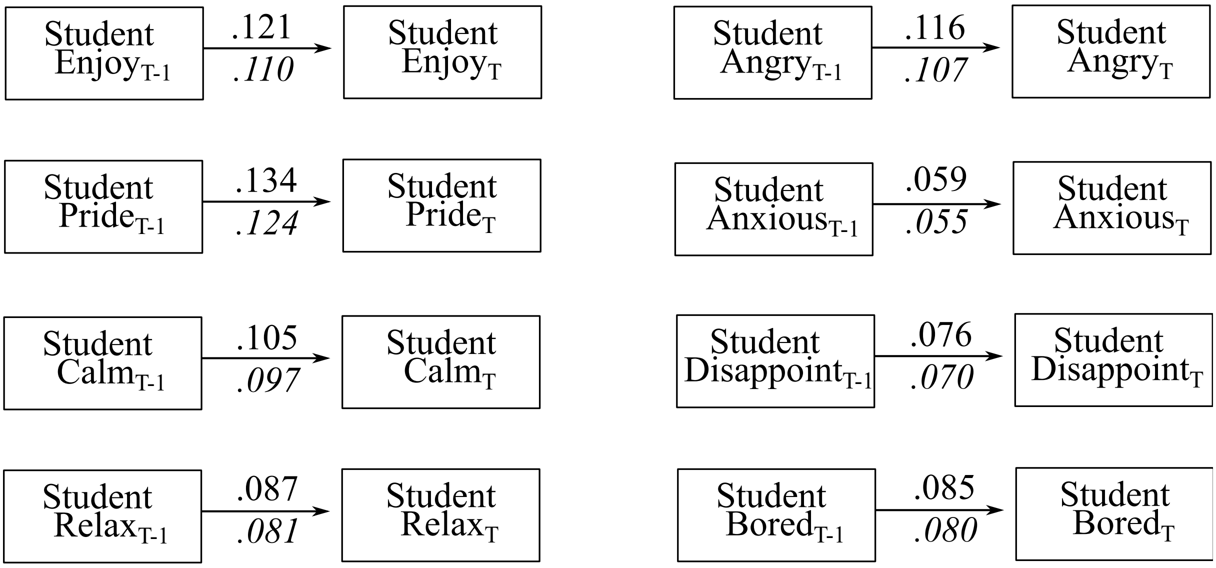

Figure 2. Auto-regressive models for discrete emotions.
Note: Unstandardised coefficients (B) above the path, and standardised coefficients (β) below. Only within-level estimates shown.

3. Discreet versus continuous time-models

While Mplus offers the possibility to specify latent lagged variables (which are latent mean centred) and to correct for non-equidistant time-lags in a two-level model (e.g., timepoints nested in students) it is not (yet) possible to use this procedure in the cross-classified or a three-level model. In order to investigate whether there would be a discrepancy between the results reported in the article based on the cross-classified models, and models in which the time-lag could be adjusted for, we specified a two-level model using the TINTERVAL and LAGGED commands included for estimating DSEM

in Mplus. We set the TINTERVAL value to one hour intervals, for time specified as elapsed time since the beginning of the study. The difference between the rank-order time (i.e., one value per lesson) and continuous time (i.e., counted in fractions of hours since the beginning of the study), is shown in Fig 3. We ran a DSEM corresponding to the cross-classified model presented in Fig 4A in the article, and largely replicated the substantive results, as shown in Table 3.

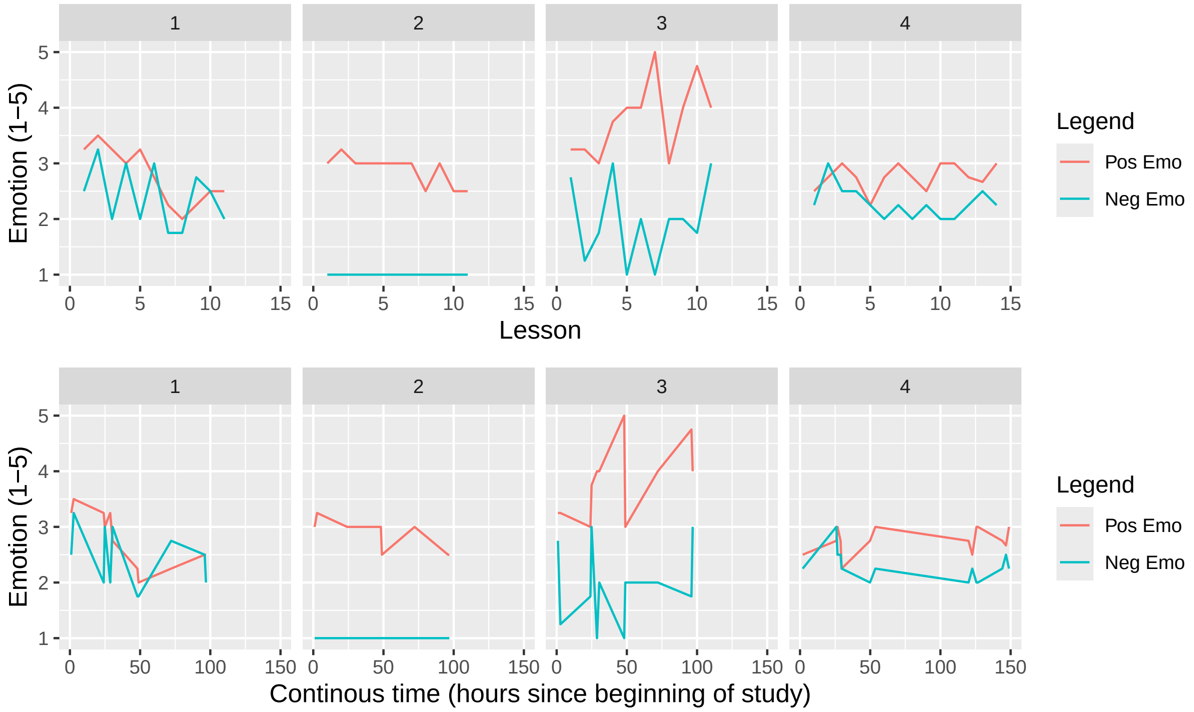

Figure 3. Positive and negative emotions of four example students.
Note: The metric on x-axis in the upper part is in discrete time, i.e., from the first to the nth lesson. The metric on x-axis in the lower part is in continuous time (hours), measured in hours since the start of the study.

Table 3
Replication of model-results from a cross-classified model (4A) by specifying a DSEM.

Parameter	Cross-classified		DSEM	
	B	β	B	β
STPEMO_T ON STPEMO_{T-1}	0.109	0.099	0.099	0.099
STPEMO_T ON SPEMO_{T-1}	0.076	0.064	0.041	0.028
SPEMO_T ON STPEMO_{T-1}	0.003	0.003	0.034	0.050
SPEMO_T ON SPEMO_{T-1}	0.184	0.171	0.235	0.235

Note: We specified latent lagged variables and adjusted for non-equidistant time-lags.

4. Possible mediation?

It was suggested whether students' perception of their teacher's emotions would mediate the relationship between the teacher's emotions and students' emotions. In order to investigate this mediation effect, we thought of it in two ways, as shown in Fig 4. First, we estimated the indirect effect for the teacher's lagged emotion predicting the student's current perception of the teacher's emotion (a), via the student's lagged perception predicting the student's own current emotion (b), as shown in the upper part of the figure. Then, we estimated the indirect effect of the teacher's current emotion predicting the student's concurrent perception of the teacher's emotion (a), via the student's concurrent perception predicting the student's own concurrent emotion (b), as shown in the lower part of the figure. We carried out separate models for positive and negative emotions. As indicated in the figure, we found indirect effects of current emotions, but not lagged emotions.

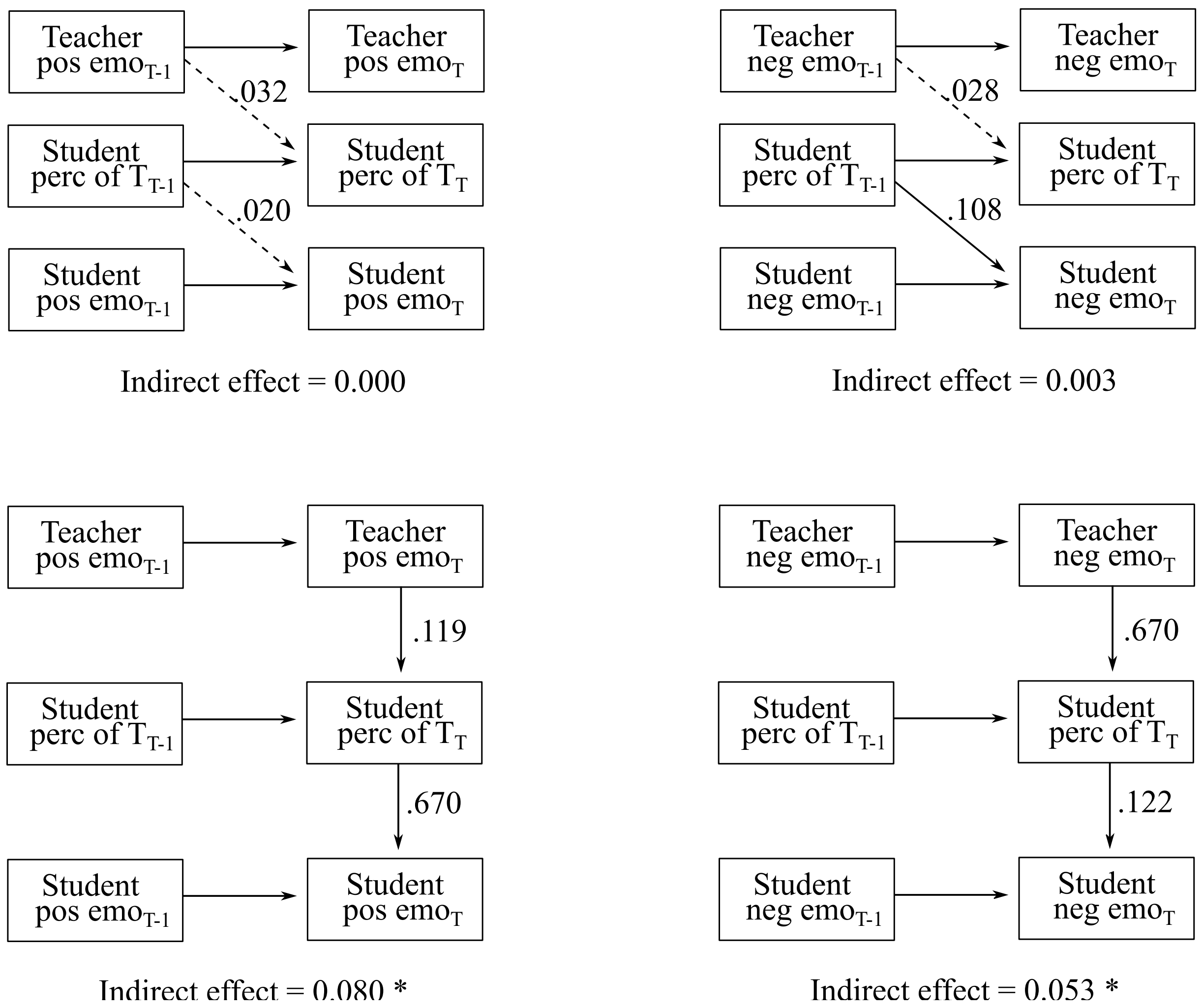

Figure 4. Mediation models for lagged (above) and current (below) emotions.
Note: Unstandardised coefficient are presented. * = indirect effect was credible

References

McNeish, D., Mackinnon, D. P., Marsch, L. A., & Poldrack, R. A. (2021). Measurement in intensive longitudinal data. *Structural Equation Modeling: A Multidisciplinary Journal*, *28*(5), 807–822. doi: 10.1080/10705511.2021.1915788

Muthén, B., & Asparouhov, T. (2012). Bayesian structural equation modeling: A more flexible representation of substantive theory. *Psychological Methods*, *17*, 313–335. doi: 10.1037/a0026802
